# ASSESSING AGE INCLUSIVITY AT THE UNIVERSITY OF HAIFA: LEADING ISRAEL’S FIRST AGE-FRIENDLY CAMPUS

**DOI:** 10.1093/geroni/igae098.1589

**Published:** 2024-12-31

**Authors:** Anna Zisberg, Orit Hirsch-Matsioulas, Sigal Naim, Adi Sapir, Yael Hochman, Chaya Koren, Lola Kolpina

**Affiliations:** University of Haifa, Haifa, Hefa, Israel; University of Haifa, Haifa, Hefa, Israel; University of Haifa, Haifa, Hefa, Israel; University of Haifa, Haifa, Hefa, Israel; University of Haifa, Haifa, Hefa, Israel; University of Haifa, Haifa, Hefa, Israel; University of Haifa, Haifa, Hefa, Israel

## Abstract

With life expectancy increase, higher awareness of ageism, and the need to reduce inequalities by ensuring that education and employment opportunities are open to all ages, the Age-Friendly University (AFU) was initiated at the University of Dublin, Ireland in 2012, and has since spread to other countries. The University of Haifa, located in a city with a higher proportion of senior citizens, compared to the national average in Israel, is leading the first AFU in Israel. We aim to present a comprehensive assessment of the prevailing campus environment and its inclusivity towards individuals of all ages. As an initial measure, we embraced the Age-Friendly Inventory and Campus Climate Survey (ICCS) questionnaire (Silverstein et al., 2022). After translating into Hebrew, we made cultural and technical adaptations and conducted validation with a panel of five experts. The survey was disseminated across the campus, garnering responses from 1500 participants. Analysis revealed generally positive attitudes towards the integration of older individuals within the campus community, although there was insufficient progress in various domains. Notably, the incorporation of ageing-related topics in teaching and research emerged as deficient. Study findings serve as a basis for developing educational programs for older learners, as well as for the reorientation of university policies towards age inclusivity. Furthermore, they provide recommendations for fostering age-friendly environments through policy formulation and organizing social-cultural initiatives on campus and beyond. In addition, the preliminary validation of the survey instrument suggests its suitability for implementation in non-English-speaking academic contexts.

